# Reassessment reveals underestimation of infiltration depth in surgical resection specimens with lymph-node positive T1b esophageal adenocarcinoma

**DOI:** 10.1055/a-2509-7208

**Published:** 2025-02-05

**Authors:** Man Wai Chan, Esther A. Nieuwenhuis, Sybren L Meijer, Marnix Jansen, Michael Vieth, Mark I. van Berge Henegouwen, R. E. Pouw

**Affiliations:** 1 1209 Gastroenterology and Hepatology, Cancer Center Amsterdam, Amsterdam Gastroenterology Endocrinology Metabolism, Amsterdam UMC Locatie VUmc, Amsterdam, Netherlands; 2 26066 Pathology, Amsterdam UMC Locatie AMC, Amsterdam, Netherlands; 3 8964 Pathology, University College London Hospitals NHS Foundation Trust, London, United Kingdom of Great Britain and Northern Ireland; 4 192358 University College London Cancer Institute, London, United Kingdom of Great Britain and Northern Ireland; 5 15019 Histopathology, Klinikum Bayreuth GmbH, Bayreuth, Germany; 6 26066 Surgery, Amsterdam UMC Locatie AMC, Amsterdam, Netherlands

**Keywords:** Endoscopy Upper GI Tract, Barrett's and adenocarcinoma, GI surgery, GI Pathology

## Abstract

**Background and study aims:**

Endoscopic resection (ER) has proven effective and safe for T1 esophageal adenocarcinoma (EAC). However, uncertainty remains concerning risk-benefit return of esophagectomy for submucosal lesions (T1b). Surgical series in past decades have reported significant risk of lymph node metastasis (LNM) in T1b EAC, but these rates may be overestimated due to limitations in histological assessment of surgical specimens. We aimed to test this hypothesis by reassessing histological risk features in surgical specimens from T1b EAC cases with documented LNM.

**Patients and methods:**

A retrospective cross-sectional study (1994–2005) was conducted. Patients who underwent direct esophagectomy without prior neoadjuvant therapy for suspected T1b EAC with LNM were included. Additional tissue sections were prepared from archival tumor blocks. A consensus diagnosis on tumor depth, differentiation grade, and lymphovascular invasion (LVI) was established by a panel of experienced pathologists.

**Results:**

Specific depth of submucosal invasion (sm1 to sm3) was not specified in 10 of 11 archival case sign-out reports. LVI status was not reported in seven of 11 cases. Following reassessment, one patient was found to have deep tumor invasion into the muscularis propria (T2). The remaining 10 of 11 patients exhibited deep submucosal invasion (sm2–3), with five showing one or more additional risk features (poor differentiation and/or LVI).

**Conclusions:**

Our findings highlight the potential for underestimating tumor depth of invasion and other high-risk features in surgical specimens. Despite the limited cohort size, our study confirmed a consistent high-risk histological profile across all cases. Caution is warranted when extrapolating LNM risk data from historic heterogeneous cross-sectional surgical cohorts to the modern ER era.

## Introduction

Significant advance in therapeutic endoscopic techniques have ushered in a paradigm shift in treatment of T1 esophageal adenocarcinoma (T1 EAC) in recent decades. The approach to treating T1 EAC has notably evolved from traditional surgical methods to adoption of endoscopic resection (ER) procedures 1 2 . To determine the most appropriate treatment strategy for a patient with T1 EAC, it is necessary to assess risk of lymph node metastases (LNM), which is associated with histological risk factors such as tumor invasion depth, differentiation grade, and lymphovascular invasion (LVI).

For patients with T1 EAC confined to the mucosa (T1a) without histologic risk factors such as poor differentiation and/or LVI, risk of LNM is minimal (< 1%). Therefore, the majority of centers worldwide have accepted ER for this indication as the primary treatment modality replacing esophagectomy. This transition is favorable, because it avoids the high complication rates associated with esophagectomy 2 3 . In case tumor invasion reaches into the submucosal layer (T1b) of the esophagus, esophagectomy with lymph node dissection is often still recommended, based on concerns over risk of LNM 4 5 6 . This recommendation is based on surgical literature, which reported LNM in T1b EAC in up to 46% 7 8 . In contrast, studies reporting LNM in T1b EAC patients treated with less invasive ER have presented a considerably lower LNM risk for T1b EAC, ranging from 0% to 26%, with higher rates in deeper invading cancers (sm2/3), and/or with additional histological risk factors such as poor differentiation and LVI 9 10 11 12 . These LNM rates are based on endoscopic follow-up data, as well as on studies in which patients underwent surgery after R0 ER of T1b EAC.

**Fig. 1:**
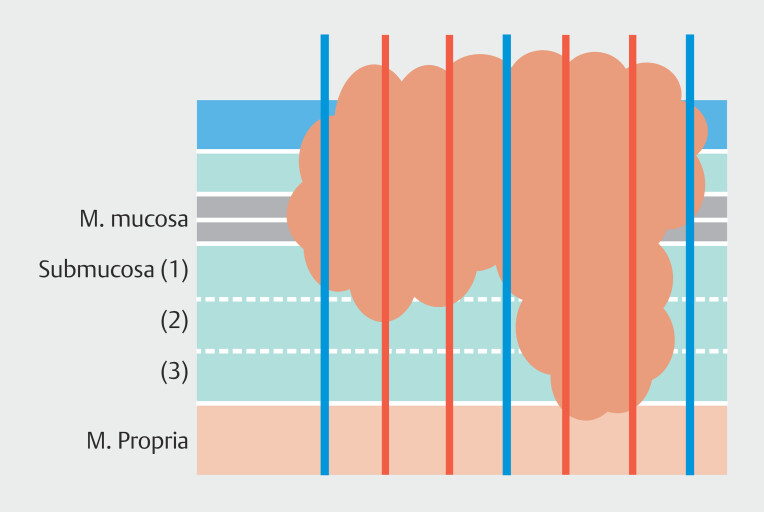
Schematic diagram illustrating a malignant tumor with invasion into the muscularis propria of the esophagus (T2). The blue lines represent histological assessment cuts on a surgical specimen, revealing submucosal invasion. In contrast, the yellow lines represent smaller cuts that would typically be made in an endoscopic resection specimen to reveal T2 invasion.

Given these observed discrepancies in published LNM risk between the surgical and endoscopic literature, we hypothesized that one of the explanations may be that LNM rates for T1b EAC may have been overestimated in surgical series due to differences in histopathological assessment. First, the surgical studies were conducted during an era when subtyping T1b EAC into sm1, sm2, and sm3 was not routine practice, because it did not bear clinical consequences. Moreover, pathological assessment of surgical specimens might allow less detailed assessment with regard to tumor infiltration depth when compared with ER specimens. This putative disparity might arise from sectioning surgical specimens into relatively wider slides, which may inadvertently lead to underestimating tumor invasion depth and potential oversight of other histological risk factors (as illustrated in Fig. 1 ).

We aimed to test this hypothesis by reassessing histological risk factors, currently used in risk stratification, in surgical specimens of cases with lymph node-positive T1b EAC.

## Methods

### Study design

This study utilized a cross-sectional design based on data extracted from the Nationwide Network and Registry of Histo- and Cytopathology in the Netherlands (PALGA Foundation). The inclusion period spans from 1994 to 2005, with gradual integration of endoscopic mucosal resection (EMR) into clinical practice starting in 2001 and becoming standard practice after 2005. This historical cohort was selected to maximize patient inclusion, given the limited cases of surgical resection for this indication during the specified period, and to have long-term follow-up outcomes available. From the database, patients with T1b EAC and LNM treated at Amsterdam UMC location AMC were identified. Patients were included if the diagnosis was based on surgical resection specimens, in patients without prior ER and/or neoadjuvant chemotherapy/radiotherapy.

### Histological reassessment

For each included case, pre-existing 5-mm tumor slides of the surgical specimens were retrieved from the archive and evaluated by an expert gastrointestinal pathologist (SM) to select the slide with deepest tumor invasion. Additional 5-µm slides were prepared from the relevant slide and stained with hematoxylin and eosin (H&E). Consequently, the slides were digitized for reassessment, anonymized, and stored on a secure server (Philips IntelliSite Pathology Solution 3.2).

An international panel consisting of three experienced gastrointestinal pathologists (SM, MJ and MV) was formed. All three pathologists have extensive experience in assessing Barrett’s neoplasia. A consensus meeting was convened where the reference panel collectively established consensus diagnoses for all cases. Tumor reassessment was done, according to the World Health Organization classification for tumor grading 13 , for:

1. Infiltration depth; classified as invasion into the submucosa (T1b; sm1 [< 500 µm], sm2/3 [≥ 500 µm]) or muscularis propria (T2);
2. Differentiation grade; divided into well (G1), moderate (G2), poor (G3), or no differentiation (G4);
3. Presence or absence of lymphatic and/or vascular invasion (LVI).

The panel was blinded to patient, treatment, and documented pathology characteristics.

### Study endpoints

The primary endpoint of this study was the number of cases upstaged from T1b to T2 invasion after reassessment. Secondary endpoints included presence of other histological risk factors (i.e. G3–4 and/or LVI) after reassessment, and disease-specific mortality as clinical outcome.

## Results

### Historic assessment of tumor infiltration and risk features

**Fig. 2:**
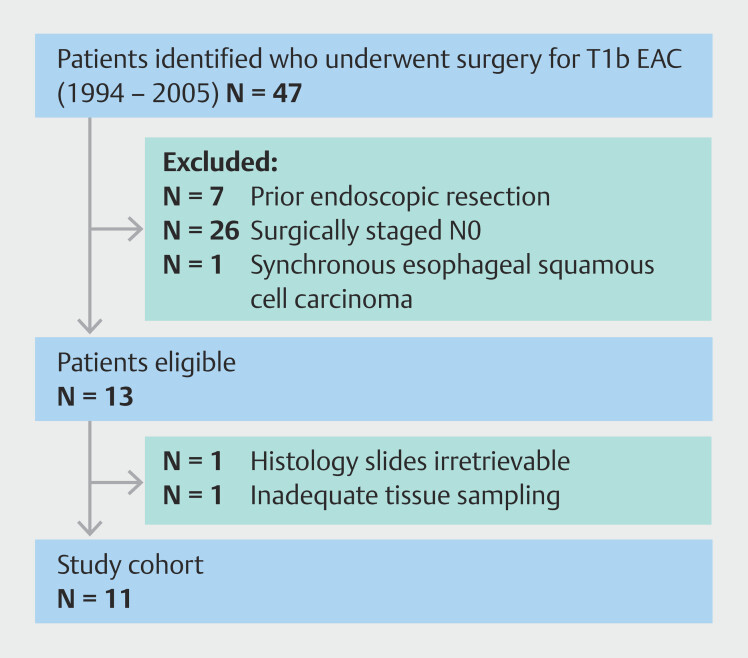
Flow of patient selection for study cohort.

Between 1994 and 2005, Amsterdam UMC location AMC treated a total of 47 patients with surgery for pT1b EAC. Among them, 13 patients were diagnosed with LNM and had not undergone prior ER or neoadjuvant chemotherapy/radiotherapy, meeting the study inclusion criteria. However, for one case, pathology slides were irretrievable from the archives. Another patient was excluded during the revision process due to inadequate tissue samples caused by sectioning artifacts and lack of full-face assessment. All reasons for and numbers of exclusion are demonstrated in Fig. 2 .

Eleven cases were included, all male, with a median age of 68 years (interquartile range [IQR] 61–72) at time of surgery. Ten of 11 patients (91%) underwent transhiatal esophagectomy and one of 11 (9%) underwent transthoracic esophagectomy. Median number of resected lymph nodes was six (IQR 5–14). Diagnoses based on the histological resection specimens are listed in Table 1 . Only in one case, the specific depth of submucosal invasion was specified (case 8; sm2). In five of 11 cases (45%), besides submucosal invasion, at least one additional histopathological risk feature was present (i.e. either ≥ G3 a/o LVI). However, LVI status was not explicitly reported in seven cases.

**Table:** **Table 1** Overview of 11 cases with surgically resected metastatic T1b EAC.

Case	Year of surgery	Surgical procedure	LNs positive/ dissected	Clinical outcome	Reported diagnosis	Revised consensus diagnosis (expert panel)
1	2002	Transhiatal	5/19 (N2)	Recurrence-free follow-up	T1sm G2 LVI+	T1sm3 G2 LVI+
2	2000	Transhiatal	3/5 (N1)*	Disease-specific death (surgical complication)	T1sm G3 LVI+	T1sm3 G3 LVI+
3	1996	Transhiatal	1/3 (N1)	Recurrence-free follow-up	T1sm G2 LVI not reported	T1sm2 G2 LVI+
4	2000	Transhiatal	3/5 (N2)	Recurrence-free follow-up	T1sm G2 LVI not reported	T1sm3 G2 LVI-
5	2002	Transhiatal	1/6 (N1)	Disease-specific death (metastatic disease)	T1sm G2 LVI not reported	T1sm3 G2 LVI-
6	1994	Transhiatal	2/6 (N1)	Disease-specific death (metastatic disease)	T1sm G2 LVI not reported	T1sm2 G2 LVI-
7	1999	Transthoracic	1/7 (N1)	Recurrence-free follow-up	T1sm G2 LVI+	T1sm3 G3 LVI-
8	1999	Transhiatal	8/14 (N3)	Disease-specific death (metastatic disease)	T1sm G3 LVI+	T1sm2 G2 LVI+
9	1995	Transhiatal	1/14 (N1)	Disease-specific death (metastatic disease)	T1sm G3 LVI not reported	T2 G2 LVI-
10	2005	Transhiatal	1/3 (N1)	Recurrence-free follow-up	T1sm2 G2 LVI not reported	T1sm2 G3 LVI-
11	1999	Transhiatal	1/10 (N1)	Recurrence-free follow-up	T1sm G2 LVI not reported	T1sm2 G2 LVI-
*Adhering to the AJCC 5 ^th^ Edition Cancer Staging Fifth edition (1997).						

### Reassessment of tumor infiltration and risk features

Consensus diagnoses, including assessment of the additionally prepared slides, showed discrepancy in nine of 11 cases with the initial pathology diagnoses of the surgical resection specimen following standard pathology procedures of that time. Histological details are provided in Table 1 .

#### Primary endpoint

**Fig. 3:**
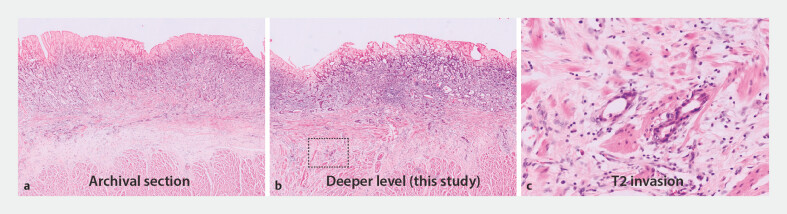
**a** Hematoxylin and Eosin (H&E)-stained surgical slide (5-mm cut) of an esophageal adenocarcinoma with submucosal invasion. **b,c** Additional cuts (5 µm) revealing invasion into the muscularis propria for the same case.

One of 11 cases was found to have T2 tumor infiltration, agreed by all three pathologists. Detailed histological images of this case are provided in Fig. 3 . The remaining 10 cases were all diagnosed with deep submucosal invasion (sm2–3).

#### Secondary endpoints

**Other histological risk factors for LNM**

In six of 11 cases, at least one additional risk feature for LNM was identified ( Table 1 ). Specifically, two cases showed an upgrading of tumor differentiation from G2 to G3, while two other cases were downgraded from G3 to G2. Tumor differentiation remained unchanged in the remaining cases. Regarding LVI, the vast majority of cases that were initially reported with LVI maintained this diagnosis upon reassessment, with one exception. In addition, one case reported as lacking LVI was shown to demonstrate LVI during reassessment (case 3).

**Disease-specific mortality**

After surgery, patients underwent clinical follow-up during median 36 months (IQR 11–52), measured until the last recorded hospital contact moment, date of recurrent event, or date of death. At the time of data collection, all 11 patients had passed away. Five patients died due to disease-related causes: one of five patients died in-hospital due to surgical complications; four of five patients died due to recurrent disease during follow-up, diagnosed median 18 months (IQR 7–33) after surgery. This subset included the patient whose revision revealed an upstaged tumor invasion to T2. The other three cases were all classified as moderately differentiated T1b tumors with deep submucosal invasion (sm2–3) and one patient also had LVI ( Table 1 ).

## Discussion

Although a number of retrospective studies have demonstrated that endoscopic follow-up in selected patients with T1b EAC is a safe approach 10 11 14 15 , there is still an ongoing discussion about this approach with some advocating surgery for this indication. In this discussion, surgical proponents often cite LNM risks of up to 46% to favor surgical esophagectomy with lymph node dissection 7 8 9 . However, we feel that these high rates of LNM in surgical literature may be an overestimation due to potential underreporting of invasion depth, due to less accurate histological assessment, and by collating all cases with submucosal invasion into a joint T1b category without differentiating between superficial and deep submucosal invasion. To test this hypothesis, this study focused on reassessment of surgical specimens in lymph node-positive T1b EAC cases.

This study was the first to reassess T1b EAC specimens directly treated by surgery by preparing additional cuts to assess the deepest point of invasion, to mimic histological assessment as currently done for ER specimens. Reassessment indeed demonstrated invasion into the muscularis propria (T2) in one case, and in addition, deep submucosal invasion (sm2/3) in all other cases. Furthermore, in addition to deep invasion, six cases demonstrated at least one other histological risk factor for LNM, such as LVI and/or poor differentiation. Even though this is a highly selected high-risk cohort with cases preceding introduction of ER and standardized neoadjuvant regimens (e.g., FLOT and CROSS protocols 16 17 ), and situated in an era where transhiatal surgery predominated, substantial disease-specific mortality observed in almost half of our patients (5 of 11) highlights the inconsistency of surgery as a definitive cure for T1b EAC patients. In the current era of patient-tailored management strategies, it is crucial to weigh this knowledge alongside morbidity scores, which can be as high as 60% even in high-volume centers, in the shared decision-making processes with patients 18 .

Although today there is no reliable prediction model available to gauge risk of LNM based on clinical and histological characteristics, it is established that risk is related to presence of submucosal invasion depth, LVI, and poor differentiation, especially if multiple risk factors are present 19 20 . The differential impact of these risk factors remains to be determined. Older studies, when assessing LNM risk through surgical specimens, typically did not report separately on presence of different risk factors, nor was the exact submucosal invasion depth reported 21 22 . Furthermore, in these historical surgical cohorts, where transhiatal esophagectomy was the prevailing procedure with a limited lymph node harvest, there might have been potential underestimation of the rate of LNM. Taking all this into account, it is currently acknowledged that these cohorts are too heterogeneous, and such studies should, therefore, be disregarded when discussing the risk-return benefit of surgery in patients with T1b EAC, especially those treated for low-risk T1b EAC. In more recent studies examining surgical specimens for LNM risk, authors commonly carry out histopathological revision of tumors in surgical resection specimens, subdividing into depths of invasion (sm1-sm3) and considering additional risk factors 20 23 . However, these studies do not involve additional deeper cuts to mimic assessments of ER specimens, potentially missing the deepest point of invasion or overlooking additional risk factors.

Although reassessment did corroborate our hypothesis in one case, we acknowledge that the number of reviewed cases to rigorously assess our hypothesis was very small. Also, we only included patients with LNM and not T1b cases without LNM, which could have increased the study cohort. However, because there are multiple research efforts ongoing in the field of watchful waiting after ER of T1b EAC, we felt that any effort to enlarge the reassessment cohort by identifying more cases and including cases without LNM, preparing additional tissue cuts and revision by an expert pathology panel, would not be appropriate. Given the limited evidence supporting our hypothesis, an alternative and plausible explanation for the disparity in LNM rates might be attributed to a benign historical selection bias. As the field has progressively matured, studies on this topic have gained increased attention and gradually transitioned to higher quality, potentially influencing reported rates of LNM. Another explanation of better outcomes after endoscopic treatment of T1b EAC in recent literature may be found in the fact that high-resolution endoscopy and virtual chromoendoscopy enables endoscopists to identify more subtle T1b lesions. In the past, these lesions were more likely to be identified at a more endoscopically visible and therefore more advanced stage. The current studies may have included a more favorable group also including earlier stage T1b lesions, compared with older series that were more likely to over-represent more advanced T1b lesions.

## Conclusions

We advocate for a more nuanced discussion regarding the decision-making process between initiating endoscopic follow-up and opting for additional surgery in selected patients diagnosed with T1b EAC. There are compelling reasons to think that LNM rates derived from surgical specimens may be an overestimation of true risk of LNM in existing reports, due to limitations of histological assessment and selection bias due to inclusion of more advanced T1b cases. This perspective gains support from recent findings in endoscopy-focused studies, which employ more precise histological assessment techniques and report LNM rates in a lower percentage of patients. As ongoing large prospective studies (PREFER trial; NCT03222635) progress, they are expected to provide more accurate and reliable LNM rates that will contribute to a better-informed understanding of optimal management strategies for T1b EAC patients.
